# On the Self-Structuring Antenna

**DOI:** 10.3390/s20030759

**Published:** 2020-01-30

**Authors:** Katarzyna Jagodzińska

**Affiliations:** Department of Electronics and Computer Science, Koszalin University of Technology, 75-453 Koszalin, Poland; katarzyna.jagodzinska@tu.koszalin.pl

**Keywords:** adaptive antenna, self-structuring antenna, reconfigurable techniques, genetic algorithm, measurements

## Abstract

This paper shows the results of an investigation on the self-structuring antenna. This antenna consists of a combination of planar paths interconnected by controllable switches. A numerical electromagnetics code (NEC) environment and application with implemented genetic algorithms were used in this research. As a result of the investigation, an antenna template was built and measured.

## 1. Introduction

As long as radiocommunication systems are evolving, parameters of the device and the wireless system determine the quality of the data transmission. Therefore, the wireless device is expected to be beam steered, multifunctional and work in a wide-band. These demands can be fulfilled with antennas that can be dynamically rearranged in response to their environment. Such antennas are called “self-structuring”, reconfigurable or adaptive.

The term “self-structuring” means that an antenna can adapt to the surroundings by changing its frequency, polarization or radiation characteristic [1,2,3]. This change redistributes the antenna currents, and thus the antenna can fit the actual environmental conditions. Thus, the self-structuring ability has become an important and desired attribute of modern, agile RF systems for sensing and wireless communications. What is more, incorporating reconfigurable antennas into wireless devices makes it smart and cognitive (able to sense the RF surrounding and communicate at the same time). Thus, reconfigurable antennas are welcome in MIMO systems, cognitive radio or satellite communication. With these smart devices, hardware complexity and cost can be significantly reduced.

Self-structuring antennas were first studied in 1983 by Schaubert [4]; then in 1999, the Defense Advanced Research Projects Agency (DARPA) launched a program under the name Reconfigurable Aperture Program to investigate reconfigurable antennas and their potential applications [5]. From that moment, many reconfigurable antennas had been investigated and designed [6,7,8,9,10,11,12,13,14,15,16].

To make an antenna reconfigurable, various techniques can be applied. These techniques are based on the following:

- using photoconductive switching elements [11];
- structural alteration [12];
- using smart materials (ferrites, liquid crystals) [13];
- changing electrical size of the antenna [3,6,7,14,15,16].

Among the mentioned techniques, a changing electrical antenna size technique is the most popular. The change of electrical size is done by controlling electrical connections among the components of the antenna template. This control relies on electronic switching components (RF-MEMs, PIN diodes or varactors) to redistribute the surface currents and alter the antenna radiating structure topology. The major advantages of such components are their good isolation, and their integration into the antenna structure is easy and offers a multiplicity of antenna configurations.

This paper presents the design and measurements of the planar self-structuring antenna template. The template consists of planar paths interconnected by controllable switches that give a multiplicity of antenna configuration and make the antenna versatile.

## 2. Antenna Design

### 2.1. Antenna Template

**Figure 1:**
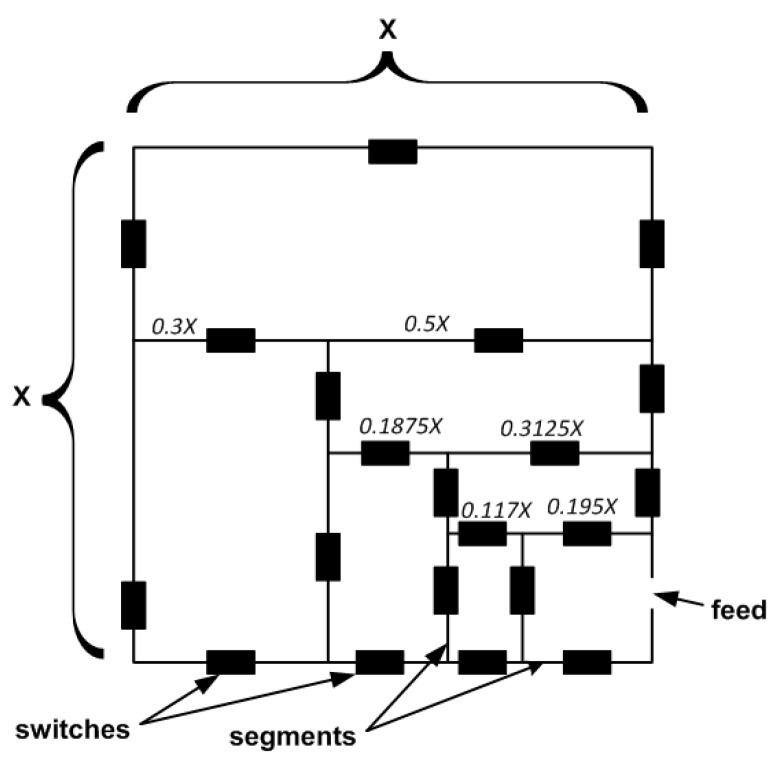
Antenna template.

For the investigation, a square shape antenna template was assumed (see Figure 1). The skeleton of an antenna had a lattice structure with planar paths (segments) interconnected by 21 controllable switches that gave 2^21^ independent configurations. The state (on/off) of the switch was controlled by a microprocessor. Assuming that the edges of the antenna were X long (X is an antenna size), the first internal segments were divided into the ratio 5/8X and 3/8X, while the next internal rectangles in the radiating lattice were unequally divided with the ratio 3/5. Such a method of division made the structure of radiating elements somehow similar to fractal.

The antenna structure and arrangement of switches allow for the creation, by optimization, of not only simple antennas such as the asymmetrical dipole or loop, but also of multi-element antennas consisting of one active element and several passive elements.

### 2.2. Antenna Simulation

As the antenna skeleton contains 21 switches, it is obvious that simulation of 2,097,152 possible configurations makes no sense, so an application based on a genetic algorithm was created to automate the simulation process [17]. With this application, an antenna template can be created and its structure written into an input NEC (numerical electromagnetic code) file. With NEC, antenna parameters (gain, *VSWR*, etc.) are found and the output file is saved. Next, the output file is used as input data for a genetic algorithm (GA) [18]. With GA, efficient (low possible *VSWR*) antenna configurations are found and saved. 

**Figure 2:**
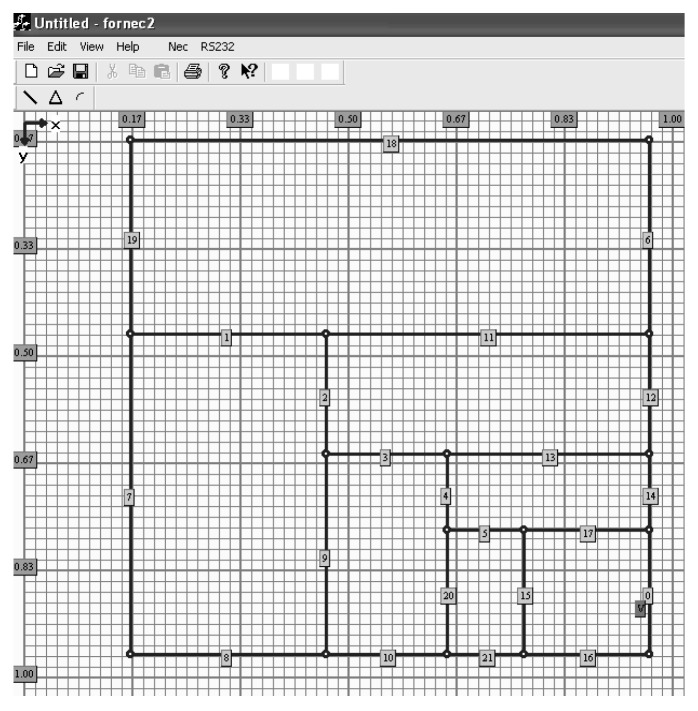
The main window of an application.

The main window of application with the defined antenna template is shown in Figure 2. The ordinate and abscissa axes are scaled in wavelength.

For simulation purposes, a center frequency was set to 800 MHz and the length of the antenna edge (X) was set to 0.8 λ so that the dimension of the antenna template was 29 × 29 cm.

**Figure 3:**
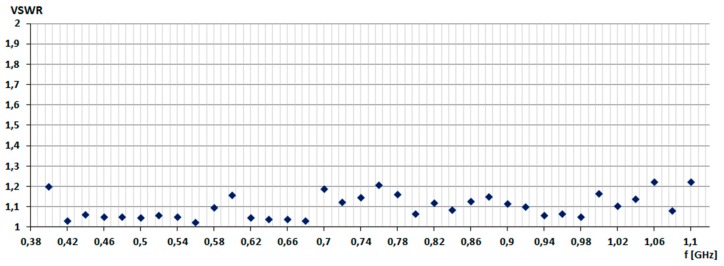
VSWR in the frequency range from 400 MHz to 1100 MHz.

The simulation aimed to determine the operating band of the antenna for frequencies in the range from 400 to 1100 MHz (see Figure 3). Simulations were performed with 40 MHz increments, repeating the simulations for each frequency three times. Parameters of the genetic algorithm were as follow: the size of population 40, 1 new random chromosome in each iteration, maximum iteration value 10, random place of cross-over. In this way, the total number of simulated antenna configurations was 1600 (40 parents chromosomes generate 40 new chromosomes in each iteration). The goal function of the GA was to minimize *VSWR*, and the graph from Figure 3 presents the average results of *VSWR*. It can be seen that in the assumed frequency range, a genetic algorithm every time was able to find an antenna configuration with low VSWR.

As the antenna template consisted of several switches, the next step in the research concerned the determination of switch failure impact on *VSWR*. The purpose of the simulation was to find the impact of permanently open (damaged) switches on *VSWR* for frequencies in the range from 400 to 1100 MHz. For tests, an antenna configuration from Figure 2 was used. This antenna was designed for 800 MHz.

In the first step of this investigation, for randomly chosen destroyed (permanently open) switches, the GA had to find antenna configuration with *VSWR* ≤ 2. Simulations were repeated three times, and Table 1 presents achieved averaged *VSWR* values. The first column contains the number of failed switch pairs or the number of the single failed switch.

Analyzing VSWR values from Table 1, it can be seen that in the worst case its value was 1.35, while the best value was 1.25. Comparing the presented VSWR values with the VSWR of the full-working antenna, the results show that the value of this parameter did not change significantly. The average value of the *VSWR*, taking into account all the examined cases, was 1.28. The *VSWR* value for the non-destroyed antenna (all switches were working) was 1.10, so switch failure did not affect antenna parameters while working on designed frequency. 

**Figure 4:**
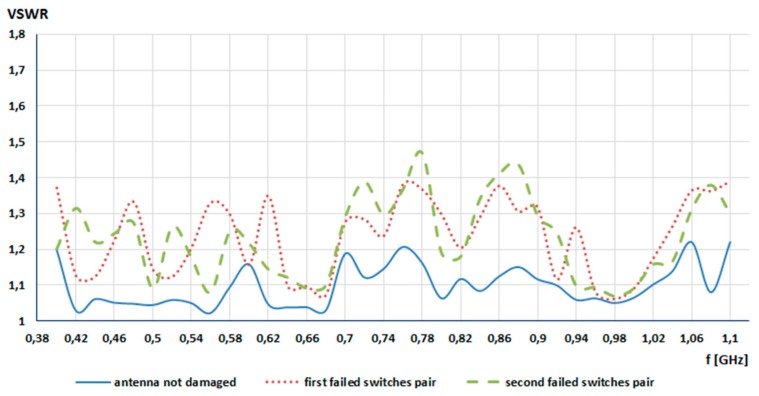
VSWR stability in the frequency range from 400 MHz to 1100 MHz.

In the second step of this research, for a given pair of failed switches, a switch failure impact on *VSWR* stability in the given frequency range was investigated. In this case, two pairs of switches were damaged (open). The simulation started at 400 MHz with a step of 20 MHz and ended at 1100 MHz. The goal function of GA was the same as before (*VSWR* ≤ 2), and the antenna from Figure 2 was used. The simulation was repeated three times and the results obtained were averaged. The achieved *VSWR* values in the given frequency range are shown in Figure 4. The first failed switches pair was switch no. 9 and no. 13, while the second failed switches pair was switch no. 2 and no. 15.

Taking into consideration the graph from Figure 4, it is seen that the switch failure impact on antenna VSWR was small. For the first pair of damaged switches (9, 13), the averaged *VSWR* was 1.249, and for second pair (2, 15) it was 1.251. For the full-working template, the averaged *VSWR* was 1.10, which is only 0.15 lower in comparison to the damaged template.

### 2.3. Antenna Measurements

**Figure 5:**
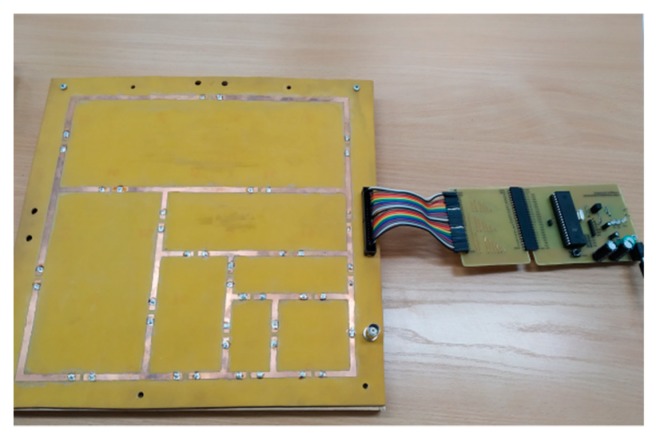
A prototype of antenna template with microcontroller.

After completing simulations, a prototype of the self-structuring antenna was constructed. The prototype is shown in Figure 5. As antenna frequency was set to 800 MHz, the size of the template was 29 x 29 cm.

The antenna template was built on FR4 (*tgδ* = 0.08, *ε* = 4.4) laminate, and the width of the antenna sections on the laminate was 4 mm. The next step was to make mounting holes in the laminate for switches (type JQC-3FF); after that, the BNC connector and the ZL231-40PG connector were mounted.

Each switch could be controlled separately with an Atmega16 microcontroller steered with a computer program. The program was written in Visual Studio and used virtual COM port software to exchange data between the computer USB port and the microcontroller USART (universal synchronous/asynchronous receiver–transmitter). Optic isolation was used in the transmission line to increase the safety of the computer and to separate the microcontroller board from the USB port.

The measurement setup consisted of an antenna tripod, signal generator series ESG Agilent Technologies (model E4432B) and field analyzer (model PROTEK 3201). The tested antenna was placed on a tripod and it was working in transmitting mode. The measurement setup was located in an open area.

To identify the measuring signal, an amplitude modulation was used. The carrier frequency was set to 1 and 1.1 GHz, modulation index was 60% and the modulating signal was 1 kHz.

**Figure 6:**
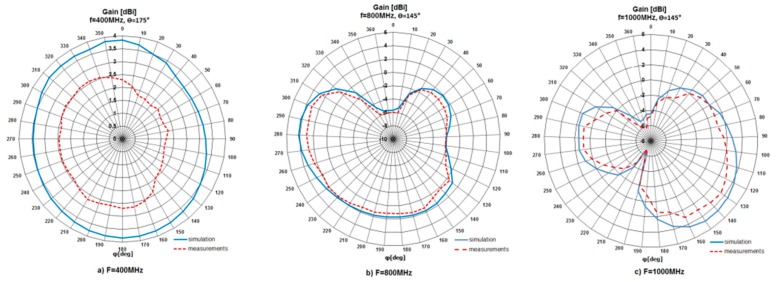
Simulated and measured radiation pattern in horizontal plane for different frequencies.

**Figure 7:**
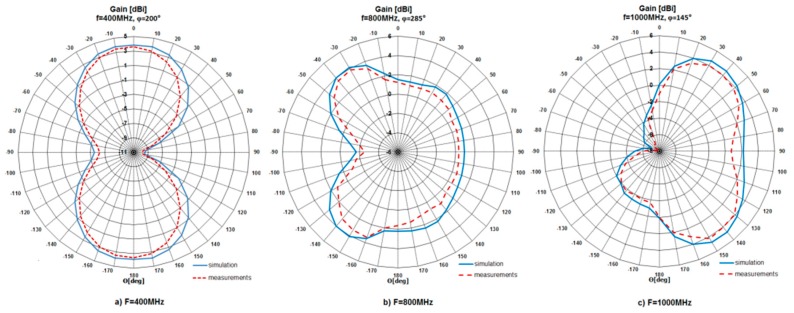
Simulated and measured radiation pattern in elevation plane for different frequencies.

After a series of measurements, gathered results were presented in the form of radiation patterns and compared with the simulation ones. The patterns were shown for three frequencies, i.e., for 400 MHz, 800 MHz and 1000 MHz in horizontal and elevation planes. The patterns are presented in Figure 6 and Figure 7, respectively. According to the achieved results, the behavior of the measured patterns was similar to the simulation ones. What is more, the measured patterns were slightly narrower than simulated. The maximum difference between patterns was 2.3 dB. The investigated antenna (for operation at 1000 MHz) had a maximum gain (*G_max_*) value equal to 4.78 dBi (simulation) and 3.83 dBi (measurement). For operation at 400 MHz, *G_max_* = 3.83 dBi (simulation) and 2.69 dBi (measurement), while for operation at 800 MHz, *G_max_* = 4.17 dBi (simulation) and 3.83 dBi (measurement).

**Figure 8:**
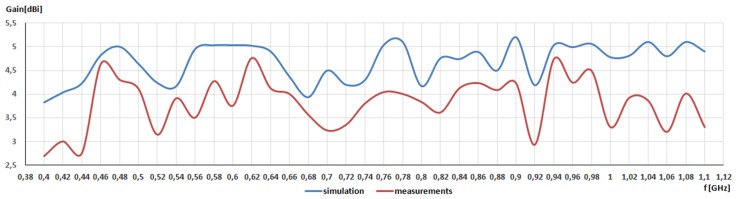
Simulated and measured gain for frequency range from 400 MHz to 1100 MHz.

In addition to radiation patterns, gain values in the frequency from 400 MHz to 1100 MHz were investigated, as seen in Figure 8. The maximum gain value was 5.1 dBi (simulation) and 4 dBi (measurement). Measured gain values lower than the simulation ones indicated the level of losses introduced by non-ideal switches. The maximum difference between simulation and measurement results was 1.48 dB.

**Figure 9:**
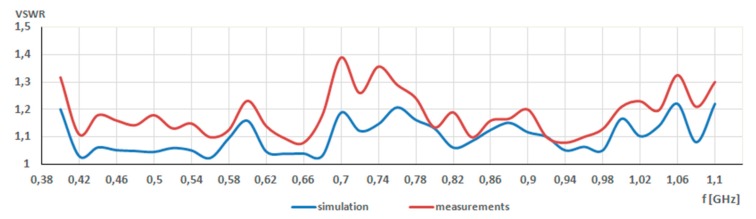
Simulated and measured *VSWR* for frequency range from 400 MHz to 1100 MHz.

The last step of measurements concerned the determination of *VSWR* values and their comparison with the values obtained during the simulation. As a result, the *VSWR* chart was achieved and it is presented in Figure 9. The simulations show that the *VSWR* values changed from 1.22 to 1.02, while measured *VSWR* changed from 1.3 to 1.05. Therefore, a good agreement between simulation and measurement results was achieved. What is more, it indicated that biasing lines of switches had a negligible impact on the antenna performance.

## 3. Conclusions

This paper dealt with an investigation of a self-structuring antenna. The self-structuring ability was achieved by using controllable switches. The state (on/off) of switches was controlled by a genetic algorithm. Every time the state of switches was changed, a new antenna configuration was achieved. In this way, the electrical size of the antenna was altered. 

It was shown that through a proper switch configuration, it is possible to find an antenna configuration with low *VSWR* (*VSWR* < 2) in a wide frequency range. What is more, it was shown that neither switch failure nor its location affects *VSWR* values. The achieved results will encouraged further work to investigate self-structuring antenna behavior, for example, in the presence of the human body (wearable antennas) or in other applications where multiband is needed.

## Figures and Tables

**Table 1:** Switch failure impact on the average VSWR value.

Failed Switch Numbers	VSWR
17, 16	1.28
15, 14	1.26
3, 12	1.30
2, 9	1.33
1, 9	1.30
9, 11	1.35
15, 17	1.31
17	1.30
15	1.26
14	1.25
12	1.26
9	1.26
3	1.25
2	1.32
1	1.28

## References

[1] Yassin A.A., Saeed R.A., Alsaqour R.A., Mukhtar R.A. (2014). Design of Reconfigurable Multi-Band Microstrip Patch Antenna with Ground Slit for WLAN and WiMax Applications. Int. J. Appl. Eng. Res..

[2] Christodoulou C.G., Tawk Y., Lane S.A., Erwin S.R. (2012). Reconfigurable antennas for wireless and space applications. Proc. Ieee.

[3] Chattha H.T., Tahir F.A., Hanif M., Sharif A., Raza A. Reconfigurable Antenna design for Portable Applications. Proceedings of the 18th International Symposium on Antenna Technology and Applied Electromagnetics (ANTEM).

[4] Schaubet D.H., Farrar F.G., Hayes S.T., Sindoris A.R. (1983). Frequency agile polarization diverse microstrip antennas and frequency scanned arrays. U.S. Patent.

[5] Smith J.K. Reconfigurable Aperture Program (RECAP)—MEMS Revolutionary Impact on RF Systems, Notes of the Workshop “RF MEMS for Antenna Applications. Proceedings of the IEEE International Symposium on Antennas and Propagation and USNC/URSI National Radio Science Meeting.

[6] Coleman C.M., Rothwell E.J., Ross J.E., Nagy L.L. (2002). Self-structuring antenna. Ieee Antennas Propag. Mag..

[7] Greetis L., Ouedraogoa R., Greetis B., Rothwell E.J. (2010). A Self-structuring patch antenna: Simulation and prototype. IEEE Antennas Propag. Mag..

[8] Hinsz L., Braaten B.D. (2014). A frequency reconfigurable transmitter antenna with autonomous switching capabilities. Ieee Trans. Antennas Propag.

[9] Tawk Y., Ayoub F., Christodoulou C.G., Costantine J. A MIMO cognitive radio antenna system. Proceedings of the 2013 IEEE Antennas and Propagation Society International Symposium (APSURSI).

[10] Ghaffar A., Li X.J., Seet B.C. Dual Frequency and Polarization Band Reconfigurable Antenna for Mobile Devices. Proceedings of the IEEE 17th International Conference on Communication Technology (ICCT).

[11] Tawk Y., Costantine J., Barbin S.E., Christodoulou C.G. Integrating laser diodes in a reconfigurable antenna system. Proceedings of the 2011 SBMO/IEEE MTT-S International Microwave and Optoelectronics Conference (IMOC 2011).

[12] Mazlouman S.J., Soleimani M., Mahanfar A., Menon C., Vaughan R.G. (2011). Pattern reconfigurable square ring patch antenna actuated by hemispherical dielectric elastomer. Electron. Lett..

[13] Hu W., Ismail M.Y., Cahill R., Encinar J.A., Fusco V.F., Gamble H.S., Linton D., Dickie R., Grant N., Rea S.P. (2007). Liquid-crystal-based reflectarray antenna with electronically switchable monopulse patterns. Electron. Lett..

[14] Jung C.W., Lee M., Li G.P., Flaviis F. (2006). Reconfigurable scan-beam single-arm spiral antenna integrated with RF-MEMS switches. Ieee Trans. Antennas Propag..

[15] Haider N., Caratelli D., Yarovoy A.G. (2013). Recent Development in Reconfigurable and Multiband Antenna Technology. Int. J. Antennas Propag..

[16] Yeole D.S., Khot U.P. Reconfigurable Multiband Microstrip Patch Antenna design for Wireless Communication Applications. Proceedings of the IEEE International Conference On Recent Trends In Electronics Information Communication Technology (RTEICT).

[17] Darnowski F., Wysota M., Jagodzińska K., Walkowiak M. Bandwidth and radiation pattern of Self structuring antennas. Proceedings of the 5th National Conference on Information Technology.

[18] Goldberg E. (1989). Genetic Algorithms in Search, Optimization, and Machine Learning.

